# Bacterial profile, their antibiotic susceptibility pattern, and associated factors of urinary tract infections in children at Hiwot Fana Specialized University Hospital, Eastern Ethiopia

**DOI:** 10.1371/journal.pone.0283637

**Published:** 2023-04-05

**Authors:** Shambel Mekonnen, Tewodros Tesfa, Tadesse Shume, Fikru Tebeje, Kedir Urgesa, Fitsum Weldegebreal

**Affiliations:** School of Medical Laboratory Sciences, College of Health and Medical Sciences, Haramaya University, Harar, Ethiopia; Leibniz-Institute DSMZ, GERMANY

## Abstract

**Background:**

Urinary tract infections (UTIs) are common pediatric infections and contribute to high morbidity and mortality. At present, the antimicrobial resistance emergency has quadrupled worldwide and poses a serious threat to the treatment of patients. However, there have been few studies on UTIs in children in Ethiopia, particularly in the east.

**Objective:**

This study aimed to assess the bacterial profile of urinary tract infections, their susceptibility to antimicrobial agents, and associated factors in under-five children at Hiwot Fana Specialized University Hospital, eastern Ethiopia.

**Method:**

We conducted hospital-based quantitative study on 332 consecutively selected under-five children from March 20 to June 10, 2021. Parents and guardians were interviewed to collect data using a structured questionnaire. Random urine samples were collected aseptically, and standard microbiological techniques were used to identify the bacteria and test for susceptibility to various antibiotics. Data were entered into Epi Info version 7 and exported to Statistical Package for the Social Sciences (SPSS) version 25 for analysis. Data were analyzed using descriptive analysis, bivariate, and multivariable logistic regression analysis. The crude odds ratio (COR) and adjusted odds ratio (AOR) with their respective 95% confidence intervals (CI) were used to determine the significance of the predictors. A p-value at a 95% confidence interval of less than 0.05 was considered statistically significant.

**Results:**

The overall prevalence of bacterial urinary tract infections was 80 (24.1%) 95% CI:19.40–29.00%). Most of the bacterial isolates 55 (68.75%) were gram-negative bacteria, predominantly *E*. *coli* 23 (28.75%) and *K*. *pneumoniae* 10 (12.50%). Being a rural resident (AOR: 4.10, 95%CI: 1.45 11.54), uncircumcised male (AOR: 3.52, 95%CI: 1.33, 9.39), previous history of antibiotic usage (AOR: 7.32, 95%CI: 2.11, 25.37), indwelling catheterization (AOR: 10.35, 95%CI: 3.74, 28.63), previous history of urinary tract infections (AOR: 5.64, 95% CI: 1.36, 23.38), and urinary frequency (AOR: 5.56, 95%CI: 2.03, 15.25) had higher odds of culture positive result. The majority of the isolates have shown high levels of antibiotic resistance. Meropenem, ciprofloxacin, and amoxicillin-clavulanic acid were effective against gram-negative uropathogens, whereas rifampin and ciprofloxacin were the most sensitive drugs for gram-positive isolates. From the tested bacterial isolates, 53/86 (61.6%), 11/86 (11.6%), and 2/86 (2.3%) were found to have multidrug resistance (MDR), extreme drug resistance (XDR), and pan drug resistance (PDR), respectively.

**Conclusions:**

About one-fourth of the children were culture-positive for many types of bacterial uropathogens; this is higher compared with most of the previous studies in Africa. Rural dwellers, uncircumcised males, indwelling catheterization, a history of antibiotic use and urinary tract infection, and frequent urination all had a higher risk of bacterial infections. Many isolates were resistant to multiple drugs, primarily beta-lactams. Urinary tract infections as well as the growth and spread of resistant bacterial pathogens should be monitor regularly.

## Introduction

Infections of the urinary tract (UTIs) are some of the most common causes of acute morbidity and chronic medical conditions like hypertension, failure to thrive, and end-stage renal disease in children [1]. UTIs, which are caused by bacteria, are a serious public health problem and a significant cause of morbidity in infant boys, older men, women of all ages, and about 5% of girls and 2% of boys by the age of seven [1]. Virulence factors used by the main uropathogens are adherence, production of the toxin, immune evasion, and iron acquisition [2]. UTIs begin when bacterial uropathogens reside anywhere in the urinary tract: the kidney, ureter, bladder, and urethra. Expression of pili and adhesins results in colonization and invasion of the superficial umbrella cells. Host inflammatory responses begin to clear extracellular bacteria, though some bacteria evade the immune system through host cell invasion, morphological changes, multiplication, and biofilm formation, as well as by producing toxins and proteases that cause host cell damage, releasing essential nutrients that promote bacterial survival and ascent to the kidneys, and progressing to bacteremia [2, 3]. The clinical manifestations of UTIs depend on the causative agent, the severity of the infection, the parts of the urinary tract involved, and the immune response of patients [4]. UTIs can be lower (cystitis) or upper (pyelonephritis) UTIs based on the patient’s clinical manifestations [5]. Serious sequelae include frequent recurrences, Urosepsis, renal scarring, progressive kidney damage in young children, preterm birth, and high-level antibiotic resistance, which can lead to high health risks, mortality, and a considerable financial burden on society [2, 6]. Globally, UTIs are common bacterial infections in humans that affect more than 150 million people and require enormous antibiotic expenditure yearly, with a global cost of more than 6 billion US dollars [7, 8]. UTIs are a common disease in developing countries, with an estimated incidence of at least 8.3 million doctor visits annually [8].

The etiologic agents of UTIs are different and usually depend on the time, geographical area, and age of patients [9]. The most prevalent bacteria causing UTIs are *Escherichia coli*, followed by *Klebsiella* spp., *Staphylococcus* spp., *Proteus* spp., *Pseudomonas aeruginosa*, *Enterobacter* spp., *Serratia* spp., *Citrobacter* spp., *Enterococcus* spp., and *Streptococci agalactiae*, with variations in their sequence of prevalence in pediatric patients [10, 11].

Several associated factors have been identified in the literature; however, the studies are not reproducible. Age, gender, premature children, and young children with severe constipation are risk factors more commonly associated with bacterial UTIs and result in a high financial burden. Other risk factors include prolonged hospitalization, congenital urinary tract obstruction, males who have not undergone circumcision, malnutrition, a previous history of UTI, urinary catheterization, and malnutrition [12–15]. The antimicrobial resistance (AMR) emergence of bacterial uropathogenic has challenged the current therapies to treat and control the spread of infections [16], and treatment has not improved and does not prevent reinfections [17]. Especially, UTIs caused by resistant bacteria are an important global medical cause of severe infections with increasing rates of morbidity and mortality [18], and they can also result in prolonged hospital stays and poverty for pediatric patients [19, 20]. In third-world countries, the problem is immense, as most healthcare facilities lack culture-based diagnostic facilities [20, 21]. Recently, multidrug-resistant (MDR) and carbapenem-resistant Enterobacterales (CREs) have emerged and gradually become a significant public health challenge [22]. Several recent studies reported the emergence of MDR bacterial uropathogens [23–25] that increase the need for the routine application of antimicrobial susceptibility testing to detect the antibiotic of choice as well as the screening of the emerging MDR strains [14, 15]. MDR, extensively drug-resistant (XDR), and pan drug-resistant (PDR) bacteria have been clearly defined according to standardized international terminology developed by the European Centre for Disease Control (ECDC) and the Center for Disease Control and Prevention (CDC), Atlanta [26]. To qualify as MDR, an agent must have gained nonsusceptibility to at least one antimicrobial agent from three or more categories. Being resistant to at least one agent in all but two or fewer antimicrobial categories is XDR (i.e., bacterial isolates remain susceptible to only one or two antimicrobial categories). Non-susceptibility to any agent in any antimicrobial category is PDR. In Ethiopia, there are limited studies available on the prevalence, antibiotic susceptibility profiles, and associated factors of UTIs in children under the age of five, and this subject has never been addressed in the study area.

## Materials and methods

### Study area and period

We conducted the study in Harar town at Hiwot Fana Specialized University Hospital (HFSUH), eastern Ethiopia, from March 20 to June 10, 2021. HFSUH is the referral teaching hospital for Health and Medical Sciences students at Haramaya University that provides healthcare services to more than 5 million people around Harar and neighboring regions.

### Study design and population

We conducted hospital-based quantitative study to assess the data and know the status of bacterial UTIs and associated factors in children hospitalized with suspected cases. All under-five children who were clinically suspected of UTIs and their parents or guardians who agreed to give consent to participate in the study were included from March 20 to June 10, 2021. Children under the age of five who had taken antibiotics within the previous 10 days of the data-collecting period were excluded. This study’s source population included all patients who visited HFSUH for a specific clinical condition diagnosis. However, during the study period, children who went to the Mother and Child Health (MCH) clinic for UTI diagnoses were regarded as the target population.

### Sample size determination and sampling technique

The sample size of 332 under-five children was determined by taking the 26.8% prevalence [27] at a 95% confidence interval (CI), a 5% margin of error, and a 10% non-respondent rate. Study participants were recruited by convenient sampling technique until we got the required sample size.

## Data and sample collection

Data on sociodemographic characteristics, clinical future, medical history, and associated factors were collected using a pretested, structured questionnaire adopted after reviewing various literature [12, 15, 28, 29] through a face-to-face interview of parents or guardians in their local languages and collecting urine samples from under-five children at the same time. The interviewer nurses performed an appropriate physical examination and card review of the child during medical registration. For toilet-trained children, a freshly cleaned random urine sample was collected in a wide-mouthed, sterile, leak-proof bottle after instructing the parents or guardians of the enrolled children to clean their genitals with soap and water. Urine samples were obtained from children who were not toilet trained by urethral catheterization, suprapubic aspiration (SPA), use of a pediatric urine collection bag, or leaving the child with the diaper off and obtaining a clean catch of urine when the child voids. Contamination was managed by giving proper instructions on how to handle the specimen correctly. The samples were delivered to the microbiology laboratory at Haramaya University College of Health and Medical Sciences within 30 minutes of collection [30], in a cold box.

### Bacterial isolation and identification

We used Chesbrough’s recommended culture and biochemical procedures for bacterial isolation and phenotypic characterizations [31]. Well-mixed urine specimens were inoculated on 5% blood agar and Cysteine Lactose Electrolyte Deficient Medium (CLED) (HiMedia, Mumbai, India) plates [5, 32], using a 0.001 ml calibrated loop. After overnight aerobic incubation at 37°C, colony morphology, gram staining, and biochemical tests were used for phenotypic characterizations [31] (supplementary material).

Identifications of gram-negative bacterial species were determined by performing a total of eleven biochemical reactions (Oxoid Ltd, Basingstoke, UK), including triple sugar iron agar (sugar fermentation, gas, H2S production, and gas), indole, urease, citrate utilization, oxidase, lysine iron agar, methyl red/Voges Proskauer (MR/VP), phenylalanine deaminase, mannitol, ornithine decarboxylase, and motility test (supplementary material). Identifications of Enterobacterales and Pseudomonas bacterial isolates were confirmed using the Analytical Profile Index (API) 20E/20NE identification kit (BioMerieux, France) [33]. However, gram-positive isolates were identified by using nine biochemical tests (Oxoid Ltd, Basingstoke, UK), including catalase, coagulase, coagulase mannitol, DNase, novobiocin sensitivity, bacitracin sensitivity, bile esculin test, urase, ornithine decarboxylase, Pyrrolidonyl arylamidase (PYR), and hemolytic characteristics on blood agar [5, 31, 32], (supplementary material).

### Antimicrobial susceptibility testing (AST)

Based on recommendations from the Clinical and Laboratory Standards Institute (CLSI) [34], modified Kirby-Bauer disk diffusion techniques were used for susceptibility testing. The suspension of bacterial inoculum, equivalent to 0.5 McFarland standards, was uniformly spread on Mueller–Hinton agar (HiMedia, Mumbai, India) plates using a sterile applicator cotton swab.

The following antimicrobial categories were tested (Oxoid, LTD, Basingstoke, and Hampshire, UK): aminoglycosides group (gentamicin (GN 10μg) and (tobramycin (TN 10μg), penicillin group (ampicillin (AMP 10μg), beta-lactamase inhibitor combination group (amoxicillin-clavulanic acid (AMC 10μg), cephalosporins class (ceftazidime (CAZ 30μg), (cefoxitin (CXT 30μg), and (cefazolin, Cz 30μg), fluoroquinolones group (ciprofloxacin (CIP 5μg), (nalidixic acid (NA 30μg), and (rifampin (RF 5μg), nitrofuran categories (nitrofurantoin (NI 300μg), competitive Inhibitors group (trimethoprim/sulphamethazole (TS 1.25/23.75μg), tetracycline (T 30μg), and phenicol (chloramphenicol (C 30μg), macrolides class (azithromycin (AZM 10 μg) and erythromycin (E 15μg), lincosamides class (clindamycin (CN 2μg), a carbapenem (meropenem (MEM 10μg), glycopeptides (vancomycin (VA 30μg), and (azetronam (ATM 30μg). These antibacterial disks were selected depending on local availability, pathogens, and 2020 CLSI recommendations. The interpretative guidelines set by the CLSI [34], were used to interpret the result as sensitive (S), intermediate (I), or resistant (R).

### Data quality control

To ensure the validity of the questionnaire, the English version was translated into local languages (Amharic and Afan Oromo), and vice versa, by separate language experts and pretested at Jugol Hospital. Data collectors (laboratory personnel and nurses) were trained regarding all stages of the data collection process. To prepare the culture media, manufacturers’ directions were followed. The sterility of culture media was checked by incubating overnight at 35–37°C without specimen inoculation. The performance of the culture medium and drug disks was checked using reference strains such as *E*. *coli* (American Type Culture Collection, ATCC 25922), *P*. *aeruginosa* (ATCC 27853), and *Staphylococcus aureus* (ATCC 25923) [34]. To perform a drug susceptibility test, the turbidity of the bacterial suspension was adjusted to the 0.5 McFarland standard.

### Method of data analysis

The data were double-entered into Epi Info version 7 and exported to Statistical Package for Social Science (SPSS) version 25 for analysis. A descriptive statistical tool was used to summarize the findings. The results were presented in words, graphs, and tables. Bivariate and multivariable logistic regression models were used to predict the relationship between dependent and independent variables. In the bivariate logistic regression model, independent variables with a P-value of less than 0.25 and clinical relevance of the variables were considered candidates for the multivariable logistic regression model [35]. The crude odds ratio (COR) and adjusted odds ratio (AOR) with their respective 95% CI were used to determine the significance of predictors. Finally, every variable with P-values less than 0.05 at a 95% confidence interval was considered statistically significant. Hosmer and Lemeshow goodness-of-fit-tests were used for adherence to multivariate logistic regression model assumptions, and a p-value > 0.05 was considered a good fit.

### Ethical considerations

An ethical review was obtained from the Haramaya University Institutional Health Research Ethics Review Committee (IHRERC) of the College of Health and Medical Sciences before conducting research. The reference number of the ethical letter was "Ref No IHRERC /027/2021)". An official letter of support was written to HFSUH. Each parent or guardian of a child received information on the study, including its goals, methods, potential risks, and benefits. The study participants were informed of their right to refuse or withdraw from the study at any time. Each study was unaffected by participants’ refusal to take part. Informed, voluntary, written, and signed parental consent was obtained from all parents or guardians of children after explaining the purpose and objective of the study. Participants’ confidentiality was strictly assured. Moreover, the positive case was reported to the attending physician or health professional. Besides, both study participants and data collectors used sanitizer and wore face masks to protect them from COVID-19.

## Result

### Socio-demographic characteristics

A total of 332 study participants were enrolled in this study. Out of these participants, 64.2% were males. The median age of the children was 2 years. Most of them, 88.3%, were older than 12 months. About 69% of children were rural dwellers. Around 41.9% of study participants had parents or guardians who were unable to read and write, and 54.8% were farmers (Table 1).

**Table 1:** Socio-demographic characteristics of under-five children at Hiwot Fana Specialized University Hospitals, eastern Ethiopia, 2021 (n = 332).

Variable	Categories	Frequency	Percent (%)
Sex	Male	213	64.2
	Female	119	35.8
Age group (in a month)	1–11	39	11.7
	12–59	293	88.3
Residence	Urban	103	31
	Rural	229	69
Educational status of parents or guardians	Unable to read and write	139	41.9
	Primary	101	30.4
	Secondary	55	16.6
	College and above	37	11.1
Occupational status of parent or guardians	Daily labor	21	6.3
	Merchant	55	16.6
	Farmer	182	54.8
	Govt employee	50	15.1
	Housewife	24	7.2

### Clinical future and medical history

About 139/213 (65.3%) of the males were circumcised. More than half of the participants were from inpatient wards. Most of the children (87.7%) did not have a previous history of antibiotic usage, 90.1% did not have a previous history of UTI, and 78.3% did not have a history or diagnosis of diabetes mellitus. About 29.5% of children have indwelling catheters. The majority of study participants did not complain about hematuria (78%), convulsion (74.4%), or urinary frequency (69.6%) during diagnosis. One-third of the children had a fever (Table 2).

**Table 2:** Clinical future and medical history of under-five children at HFSUH, eastern Ethiopia, 2021 (n = 332).

Variable	Categories	Number(n)	Percent (%)
Circumcised status of boys	Yes	139	65.3
	No	74	34.7
History of antibiotic usage	Yes	41	12.3
	No	291	87.7
Previous history of UTI	Yes	32	9.6
	No	300	90.4
Vomiting	Yes	115	34.6
	No	217	65.4
Indwelling catheterization	Yes	98	29.5
	No	234	70.5
Dysuria	Yes	95	28.6
	No	237	71.4
Abdominal pain	Yes	161	48.5
	No	171	51.5
Hematuria	Yes	73	22.0
	No	259	78.0
Convulsion	Yes	85	25.6
	No	247	74.4
Diarrhea	Yes	144	43.4
	No	188	56.6
History of Diabetes mellitus	Yes	72	21.7
	No	260	78.3
Temperature of children	< 38.5	222	66.9
	≥38.5	110	33.1
Duration of fever	≥1 week	35	10.5
	<1 week	75	22.6
Malnutrition status	Yes	140	42.2
	No	192	57.8
Patient type	Inpatient	173	52.1
	Outpatient	159	47.9
Urinary frequency	Yes	101	30.4
	No	231	69.6

### Prevalence of urinary tract infection

The overall prevalence of bacterial UTI was 24.1% (80/332; 95% CI: 19.4–29). A total of 86 bacterial isolates were identified. About 68.75% (55\80) of isolates were Gram-negative bacteria. In this study, the prevalence of UTI was 25.2% (30/119) in females, 28.2% (11/39) in the age <12 months, and 27.9% (64/229) in rural dwellers. There was also a higher frequency of UTI among those with a previous history of UTI (47.7%, 17/36), previous antimicrobial usage (43.9%, 18/39), children who had hematuria (42.5%, 31/73), uncircumcised boys (39.5%, 30/76), and catheterized patients (35.7%, 35/98).

### Bacterial profile of uropathogens

**Fig 1:**
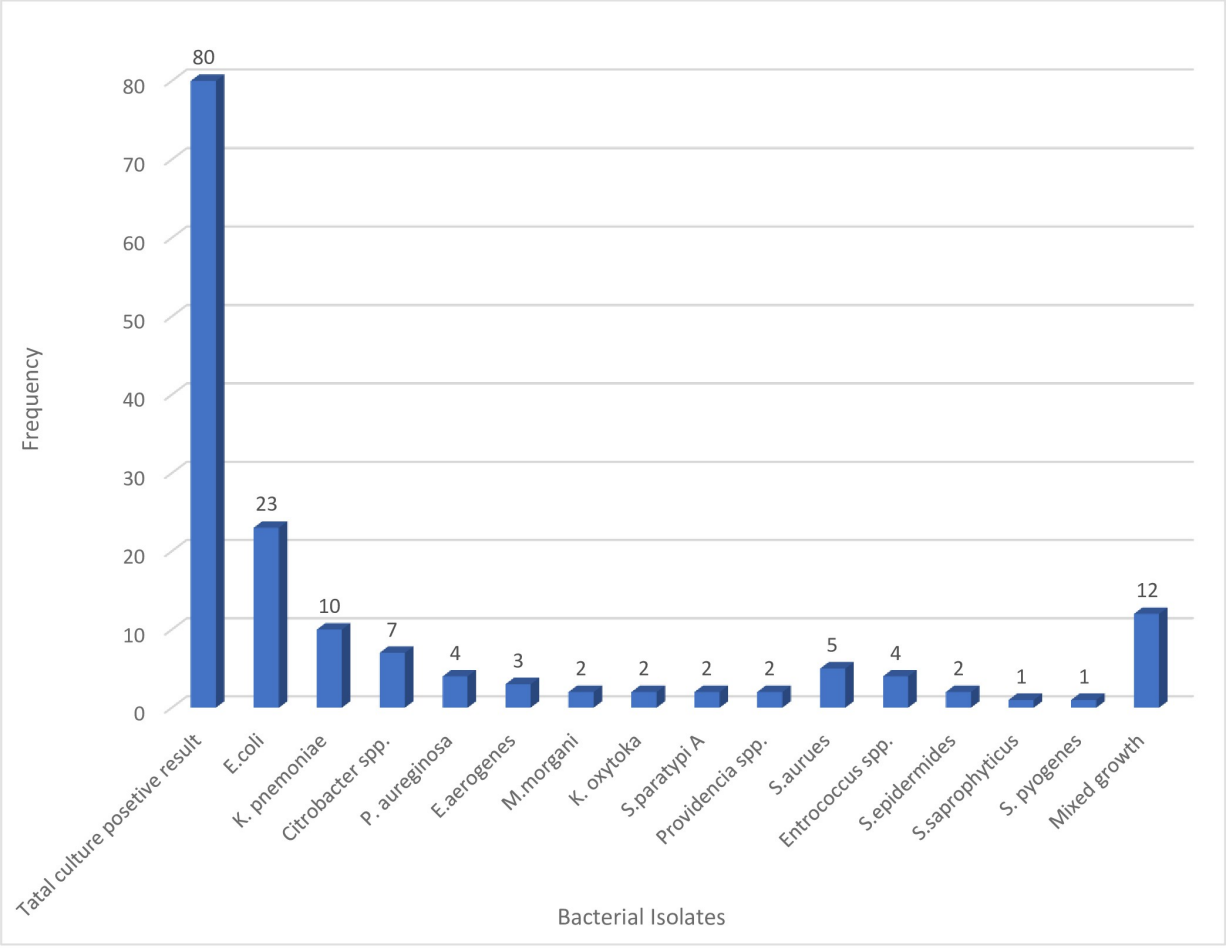
Frequency and type of bacterial isolated from the urine of under-five children at HFSUH, eastern Ethiopia, 2021.

Of the total 80 (24.1%) culture-positive bacteria, pure gram-negative bacterial species were predominant 55 (68.75%); the most frequently isolated were *E*. *coli* 23 (28.75%), followed by *K*. *pnemumoniae* 10 (12.5%). From gram-positive bacteria, *S*. *aureus* 5 (6.25%) has been isolated more commonly, followed by *Enterococcus* spp. 4 (5%), while 12 (15%) of the children had a mixed infection. Pathogens that cause mixed infections include *Staphylococcus saprophyticus*, *Citrobacter* spp., *E*. *coli*, *K*. *pnemumoniae*, *Tatumella ptyseos*, *Enterobacter aerogenes*, *Streptococcus pyogenes*, *S*almonella paratypi A, *S*. *aureus*, *Klebsiella ozaniae/Klebsiella rhinoscleromatis*, and *Enterococcus* spp. (Fig 1).

### Associated factors

In the bivariate logistic regression analysis, 12 independent variables with a p-value of less than 0.25 were candidates for the multivariable logistic regression analysis (Table 3).

**Table 3:** Factors associated with UTIs among under-five children at HFSUH, eastern Ethiopia, 2021.

Variable	Categories	UTI		COR (95% CI)	P	AOR (95% CI)	P
		Positive n (%)	Negative n (%)				
Sex	Female	30(25.2)	89(74.8)	1.1 (.653–1.850)	0.723		
	Male	50(23.5)	163(76.5)	1			
Age	<12 month	11(28.2)	28(71.8)	1.3 (.604–2.694)	0.524		
	≥12 month	69(23.5)	224(76.5)	1			
Residence	Rural	64(27.9)	165(72.1)	2.1 (1.150–3.867)	**0.016**	**4.089(1.449–11.539)**	**0.008**
	Urban	16(15.5)	87(84.5)	1		**1**	
Educational status of parents or guardians	Collage and above	6(16.2)	31(83.8)	1			
	Secondary	7(12.7)	48(87.3)	1.3 (.408–4.320)	0.26		
	Primary	26(25.7)	75(74.3)	0.6 (.209–1.490)	0.638		
	Illiterate	41(29.5)	98(70.5)	0.5 (.179–1.193)	0.28		
Occupational status of parents or guardians	Employee	9(16.7)	41(83.3)	1			
	Merchant	14(25.5)	41(74.5)	0.6 (.250–1.650)	0.359		
	Housewife	7(29.2)	17(70.8)	0.5 (.171–1.664)	0.323		
	Daily labor	6(28.6)	15(71.4)	0.54 (.167–1.804)	0.279		
	Farmer	44(24.2)	138(75.8)	0.7 (.310–1.528)	0.358		
Circumcised status of boys	No	30(40.5)	44(59.5)	3.4 (1.805–6.551)	**0.000**	**3.523(1.328–9.348)**	**0.011**
	Yes	23(16.5)	116(83.5)	1		**1**	
Past drug use without a prescription.	Yes	18(43.9)	23(56.1)	2.9 (1.468–5.692)	**0.002**	**7.315(2.109–25.368)**	**0.001**
	No	62(21.3)	229(78.7)	1		**1**	
Previous History of UTI	Yes	17(47.2)	19(52.8)	3.3 (1.625–6.738)	**0.001**	**5.638(1.360–23.376)**	**.02**
	No	63(21.3)	233(78.7)	1		**1**	
Vomiting	Yes	27(23.5)	88(76.5)	1(.558–1.614)	0.848		
	No	53(24.4)	164(75.6)	1			
Dysuria	Yes	35(36.8)	60(63.2)	2.5 (1.467–4.221)	**0.001**	.733(.254–2.116)	.566
	No	45(19)	192(81)	1		1	
abdominal pain	Yes	43(26.7)	118(73.3)	1.3 (.797–2.185)	0.281		
	No	37(21.6)	134(78.4)	1			
Hematuria	Yes	31(42.5)	42(57.5)	3.2 (1.809–5.530)	**0.000**	**2.962(1.182–7.418)**	**.02**
	No	49(18.9)	210(81.1)	1		**1**	
Convulsion	Yes	29(34.1)	56(65.9)	2 (1.155–3.429)	**0.013**	.779(.282–2.150)	.629
	No	51(20.6)	196(79.4)	1		1	
Diarrhea	Yes	36(25)	108(75)	1.1 (.658–1.810)	0.736		
	No	44(23.4)	144(76.6)	1			
History of indwelling catheterization	Yes	35(35.7)	63(64.3)	2.3 (1.379–3.947)	**0.002**	**10.35(3.742–28.63)**	**.000**
	No	45(19.2)	189(80.8)	1		**1**	
Diabetes mellitus	Yes	27(37.5)	45(62.5)	2.3 (1.333–4.121)	**0.003**	**3.694(1.303–10.476)**	**.014**
	No	53(20.4)	207(79.6)	1		**1**	
Duration of fever	> = 1 week	24(32)	51(68)	1.1 (.618–2.008)	0.719		
	<1 week	49(29.7)	116(70.3)	1			
Malnutrition status (mild, moderate, and severe)	Yes	44(31.4)	96(68.6)	2 (1.194–3.303)	**0.008**	.801(.312–2.061)	.646
	No	36(18.75)	156(81.25)	1		1	
Temperature of children	≥38.5	39(35.5)	71(64.5)	2.4 (1.446–4.068)	**0.001**	**3.995(1.544–10.331)**	**.004**
	< 38.5	41(18.5)	181(81.5)	1		**1**	
Urinary frequency	Yes	39(38.6)	62(61.4)	2.9 (1.72–4.922)	**0.000**	**5.558(2.025–15.253)**	**.001**
	No	41(17.7)	190(82.3)	1		**1**	
Patient type	Inpatient	49(28.3)	124(71.7)	1.6 (.977–2.726)	0.61		
	Outpatient	31(19.5)	128(80.5)	1			

On multivariable logistic regression analysis, being rural residents (AOR: 4.10, 95%CI: 1.45, 11.54), having uncircumcised boys (AOR: 3.52, 95%CI: 1.33, 9.39), having a previous history of UTI (AOR: 5.64, 95%CI: 1.36, 23.38), having children with past antibacterial use without prescription (AOR: 7.32, 95%CI: 2.11, 25.37), having children with hematuria (AOR: 2.96, 95%CI: 1.18, 7.42), participants having a history of indwelling catheterization (AOR: 10.35, 95%CI: 3.74, 28.63), children with diabetes mellitus (AOR: 3.69, 95%CI: 1.30, 10.48), Children with a body temperature ≥ 38.5°C (AOR: 3.99, 95%CI: 1.54, 10.33), and participants showing symptoms of urinary frequency (AOR: 5.56, 95%CI: 2.03, 15.25) were found to have a statistically significant association with UTI at a p-value less than 0.05 (Table 3).

### Antimicrobial susceptibility pattern

**Gram-negative bacteria.** The antimicrobial susceptibility test was performed on 86 bacterial isolates. About 92.7% of gram-negative isolates were susceptible to meropenem, 83.1% to ciprofloxacin, and 77.9% to amoxicillin-clavulanic acid. Additionally, 8.75% of the isolates showed sensitivity to all the antimicrobial agents used in the test panel. While, 90.5% of isolates exhibited resistance to ampicillin, 77.8% to tetracycline, 73% to cefazolin, and 72% to trimethoprim- trimethoprim-sulphamethoxazole. About 92.8% of *E*. *coli* were susceptible to meropenem, 75% to amoxicillin-clavulanic acid, and 71.4% to ciprofloxacin. However, it was 82% and 82.1% resistant to ampicillin and tetracycline, respectively (Table 4).

**Table 4:** Antimicrobial susceptibility pattern of gram-negative bacteria isolated from the urine of under-five children attending at HFSUH, eastern Ethiopia, 2021.

bacterial isolate	Pattern	Antimicrobial															
		AMP (%)	CIP (%)	GM (%)	AUG (%)	TS (%)	T (%)	CZ (%)	CAZ (%)	NI (%)	CXT (%)	MEM (%)	TN (%)	ATM (%)	NA (%)	CPM (%)	R0
*E*. *coli (n = 28)*	S	5(18)	20(71.4)	13(46.4)	21(75)	7(25)	4(14.3)	6(21.4)	9(32.1)	11(39)	14(50)	24(92.8)	8(28.6)	11(39)	16(57)	-	6
	I	0	0	2(7.1)	0	0	1(3.4)	1(3.4)	0	10(36)	1(3.4)	0	5(18)	2(7.1)	2(7.1)	-	
	R	23(82)	8(28.6)	13(46.4)	7(25)	21(75)	23(82.1)	21(75)	19(67.8)	7(25)	13(46.4)	2(7.1)	15(53.6)	15(53.6)	10(35.7)	-	
*K*. *pneumoniae (n = 13)*	S	1(7.7)	10(76.9)	3(23.1)	10(76.9)	5(38.5)	2(15.4)	2(15.4)	1(10)	5(38.5)	10(76.9)	11(84.6)	8(61.5)	4(30.7)	6(46.2)	-	
	I	0	0	1(7.7)	0	0	1(7.7)	0	1(10)	5(38.5)	0	1(7.7)	2(15.4)	2(15.4)	2(15.4)	-	
	R	12(92.3)	3(23.1)	9(69.2)	3(23.1)	8(61.5)	10(76.9)	11(84.6)	11(84.6)	3(23.1)	3(30)	1(7.7)	3(23.1)	7(53.9)	4(30.8)	-	
*Citrobacter* spp. *(n = 8)*.	S	0	6(75)	2(25)	4(50)	1(12.5)	0	1(12.5)	1(12.5)	2(25)	2(25)	8(100)	3(38)	2(25)	4(50)	-	
	I	0	1(12.5)	1(12.5)	1(12.5)	0	1(12.5)	1(12.5)	0	3(37.5)	1(12.5)	0	3(37)	1(12.5)	2(25)	-	
	R	8(100)	1(12.5)	5(62.5)	3(37.5)	7(87.5)	7(87.5)	6(75)	8(100)	3(37.5)	5(62.5)	0	2(25)	5(63)	2(25)	-	
*P*. *aeruginosa (n = 5)*	S		4(80)	3(60)	4(80)	2(40)	-	-	1(20)		2(40)	5(100)	5(100)	1(20)		4(80)	
	R		1(20)	2(40)	1(20)	3(60)	-	-	4(80)		3(60)	0	0	4(80)		1(20)	
*E*. *aerogenes (n = 3)*	S	0	3(100)	1(25)	3(100)	1(33)	0	2(67)	2(67)	2(67)	1(33)	3(100)	1(33)	1(66)	3(100)	-	
	R	3(100)	0	2(67)	0	2(67)	3(100)	1(33)	1(33)	1(33)	2(67)	0	2(67)	1(33)	0	-	
S. Paratypi A *(n = 3)*	S	0	3(100)	01(33)	3	1(33)	0	0	0	1(33)	1(33)	3(100)	1(33)	2(67)	1(33)		
	R	3(100)	0	2(67)	0	2(67)	3(100)	3(100)	3(100)	2(67)	2(66)	0	2(66)	1(33)	2(67)		
*K*. *oxytoca (n = 2)*	S	0	2(100)	0	2(100)	0	1(50)	1(50)	2(100)	2(100)	2(100)	2(100)	2(100)	0	2(100)		
	R	2	0	2(100)	0	2(100)	1(50)	1(50)	0	0	0	0	0	2(100)	0		
*M*. *morganii (n = 2)*	S	0	2	0	2(100)	1(50)	1(50)	1(50)	2(100)	2(100)	0	2(100)	2(100)	2(100)	1(50)		
	R	2(100)	0	2(100)	0	1(50)	1(50)	1(50)	0	0	2(100)	0	0	0	1(50)		
*K*.*ozaniae/K*.*rhinoscleromatis (n = 1)*	S	0	1(100)	0	1(100)	0	0	0	0	1(100)	0	0	1(100)	0	0		
	R	1(100)	0	1(100)	0	1(100)	1(100)	1(100)	1(100)	0	1(100)	1(100)	0	1(100)	1(100)		
*T*. *pytsous (n = 1)*	S	0	1(100)	1(100)	1(100)	1(100)	1(100)	1(100)	1(100)	1(100)	1(100)	1(100)	1(100)	0	1(100)		
	R	1(100)	0	0	0	0	0	0	0	0	0	0	0	1(100)	0		
*Providencia* spp.*(n = 2)*	S	0	2(100)	2(100)	2(100)	0	2(100)	1(50)	2(100)	2(100)	0	2(100)	2(100)	2(100)	2(100)		
	R	2(100)	0	0	0	2(100)	0	1(50)	0	0	2(100)	0	0	0	0		
Total	S	**6 (9.5)**	**54(83.1)**	**26(37.7)**	**53(77.9)**	**19(28)**	**11(17.46)**	**15(23.8)**	**21(30.43)**	**28(45.9)**	**33(49.3)**	**63(92.7)**	**33(49.3)**	**26(38.8)**	**36(58.1)**	**4(80)**	
	I	**0**	**1(1.5)**	**5 (7.25)**	**1 (1.6)**	**0**	**3(4.76)**	**2(3.17)**	**1(1.45)**	**17(27.9)**	**2(2.9)**	**1(1.5)**	**10(14.9)**	**5(7.5)**	**6(9.7)**	**0**	
	R	**57(90.5)**	**10(15.4)**	**38(55.1)**	**14(21.9)**	**49(72)**	**49(77.8)**	**46(73)**	**47(68.1)**	**16(26.2)**	**32(47.8)**	**4(5.8)**	**24(35.8)**	**36(53.7)**	**20(32.2)**	**1(20)**	

Keys: AMP = ampicillin; CIP = ciprofloxacin; GM = gentamycin; AUG = augmentin; TS = trimethoprim-sulphamethoxazole; T = tetracycline; CZ = cefazolin; CAZ = ceftazidime; NIT = nitrofurantoin; CXT = cefoxitin; MEM = meropenem; TN = tobramycin; ATM = azetronam; NA = nalidixic acid CPM = cefepime; R0 = susceptible to all antibiotics; S = sensitive; I = intermediate; and R = resistant.

#### Gram-positive bacteria

Among the tested antibiotics, rifampin (81.8%), ciprofloxacin (81.25%), and gentamycin (68.75%) were effective against gram-positive isolates. On the other hand, they showed resistance to tetracycline (56.2%), tobramycin (54.5%), and trimethoprim-sulphamethoxazole (54%). *S*. *aureus* was the most predominant isolate, which revealed 83.3% sensitivity to each of rifampin and ciprofloxacin. However, there is 83.3% resistance to each of the antibiotics, ampicillin and tetracycline (Table 5).

**Table 5:** Antimicrobial susceptibility pattern of gram-positive bacteria isolated from the urine of under-five children at HFSUH, eastern Ethiopia, 2021.

Bacterial isolate	Pattern	Antimicrobial												
		AMP (%)	CIP (%)	GM (%)	TS (%)	T (%)	NI (%)	CTX (%)	TN (%)	E (%)	CLN (%)	RF (%)	C (%)	R0
*S*. *aureus (n = 6)*	S	1(16.67)	5(83.33)	4(66.67)	3(50)	1(16.67)	-	4(66.67)	3(50)	3(50)	4(66.67)	5(83.33)	33(50)	1
	I	3(50)	0	0	1(16.67)	0	-	1(16.67)	0	0	0	0	0	
	R	2(33.33)	1(16.67)	2(33.33)	2(33.33)	5(83.33)	-	1(16.67)	3(50)	3(50)	2(33.33)	1(16.67)	33(50)	
*S*. *epidermidis (n = 3)*	S	1	2	2	1	1	-	1	2	1	2	3	2	
	I	1	0	0	0	0	-	1	0	0	0	0	0	
	R	1	1	1	2	2	-	1	1	2	1	0	1	
*S*. *saprophyticus (n = 2*	S	2	2	1	1	1	-	1	0	1	0	1	1	
	R	0	0	1	1	1	-	1	2	1	2	1	1	
*Enterococcus* spp. *(n = 5)*	S	5(100)	4(80)	4(80)	-	4(80)	4(80)	-	-	4(80)	-	-	4(80)	
	R	0	1(20)	1(20)	-	1(20)	1(20)	-	-	1(20)	-	-	1(20)	
*S*. *pyogenes (n = 2)*	S	2	-	-	-	-	-	-	-	1	1	-	2	
	R	0	-	-	-	-	-	-	-	1	1	-	0	
Total	S	11(61.1)	13(81.25)	11(68.75)	5(45.5)	7(43.8)	4(80)	6(54.5)	5(45.5)	10(55.6)	7(53.8)	9(81.8)	12(66.7)	1
	I	4(22.2)	0)	0	1(9)	0	0	2(18.2)	0	0	0	0	0	
	R	3(16.7)	3(31.3)	5(31.2)	5(45.5)	9(56.2)	1(20)	3(27.3)	6(54.5)	8(44.4)	6(46.2)	2(18.2)	6(33.3)	

Keys: AMP = ampicillin; CIP = ciprofloxacin; GM = gentamycin; AUG = augmentin; TS = trimethoprim sulfamethoxazole; T = tetracycline; CZ = cefazolin; CAZ = ceftazidime; NIT = nitrofurantoin; CXT = cefoxitin; R0 = susceptible to all antibiotics; MEM = meropenem; TN = tobramycin; ATM = aztreonam; NA = nalidixic acid; CPM = cefepime; S = sensitive; I = intermediate; R = resistant.

#### MDR patterns of bacterial isolates

The overall MDR rate of the isolates was 61.6% (95% CI: 51–73). A higher rate of MDR was observed in gram-negative bacterial isolates compared to gram-positives. The majority of the gram-negative isolates 47/68 (69.1%) revealed MDR. Particularly, the highest MDR was observed in *E*. *coli*, 20/53(37.7%) followed by *K*. *pneumoniae*, 9/53 (17%), *Citrobacter spp*. 8/53 (15.1%). Multidrug resistance was also observed in a gram-positive bacterial isolate, which was 6/18(33.3%). About 3/53(5.7%) of MDR was observed in *S*. *aureus* (Table 6).

**Table 6:** The pattern of MDR bacteria among under-five children at the HFSUH, eastern Ethiopia, 2021.

Antimicrobial agent	Total n (%)	MDR bacterial isolate													
		*E*. *coli*	*K*. *pneumoniae*	*Citrobacter* spp.	*P*. *aeruginosa*	*E*. *aerogenes*	S. Paratypi A	*K*. *oxytoca*	*M*.*morganii*	*K*. *ozaniae/K*. *rhinoscleromatis*	*Providencia* spp.	*S*. *aureus*	*S*. *epidermdisus*	*S*. *saprophyticus*	*Enterococcus* spp.
**GM, T, TS,**	1(1.6)	1													
**AMP, T, CAZ**	1(1.6)		1												
**GM, CAZ, ATM**	1(1.6)				1										
**TS, T, CZ, NA**	1(1.6)	1													
**AMP, TS, T, CZ**	2(3.1)	1		1											
**AMP, GM, CZ, ATM**	2(3.1)		2												
**AMP, GM, CIP, TS, CZ,**	1(1.6)	1													
**AMP, TS, T, CZ, TN**	1(1.6)	1													
**AMP, TS, T, CZ, ATM**	1(1.6)	1													
**AMP, TS, T, CZ, NA**	3(4.6)	1							1		1				
**AMP, TS, T, CAZ, ATM**	1(1.6)			1											
**AMP, GM, TS, T, ATM**	1(1.6)					1									
**AMP, GM, CIP, TS, CZ**	1(1.6)						1								
**GM, CIP, T, E, C**	1(1.6)														1
**AMP, AUG, TS, T, CZ, TN**	1(1.6)	1													
**AMP, GM, TS, T, CZ, NA**	1(1.6)	1													
**AMP, GM, TS, CZ, NI, TN**	2(3.1)		1	1											
**AMP, GM, AUG, TS, T, CZ**	2(3.1)		1			1									
**AMP, AUG, TS, T, CZ, TN**	1(1.6)			1											
**AMP, GM, TS, T, CZ, ATM**	1(1.6)							1							
**AMP, GM, CIP, TS, T, C**	2(3.1)											1	1		
**AMP, GM, TS, T, RF, C**	1(1.6)													1	
**AMP, GM, CIP, TS, T, CZ, ATM**	3(4.7)	2	1												
**AMP, AUG, GM, CIP, T, CZ, ATM**	1(1.6)	1													
**AMP, AUG, TS, T, CZ, MEM, NA**	1(1.6)		1												
**AMP, GM, AUG, TS, T, CZ, ATM**	2(3.1)			2											
**AMP, GM, CIP, TS, T, CZ, NI**	1(1.6)			1											
**AMP, CIP, TS, T, CZ, NI, ATM**	1(1.6)						1								
**AMP, CIP, TS, T, CZ, MEM, ATM**	1(1.6)									1					
**AMP, GM, CIP, TS, T, E, C**	1(1.6)											1			
**AMP, CIP, T, TN, E, RF, C**	1(1.6)											1			
≥R8	12(18.6)	8(66.7)	2	1			1								
MDR **R≥3**	**53(61.6)**	**20(37.7)**	**9(17)**	**8(15.1)**	**1(1.9)**	**2(3.8)**	**3(5.7)**	**1(1.9)**	**1(1.9)**	**1(1.9)**	**1 (1.9)**	**3(5.7)**	**1(1.9)**	**1(1.9)**	**1(1.9)**

Keys: AMP = ampicillin; CIP = ciprofloxacin; GM = gentamycin; AUG = augmentin; TS = trimethoprim-sulphamethoxazole; T = tetracycline; CZ = cefazolin; CAZ = ceftazidime; NIT = nitrofurantoin; CXT = cefoxitin; MEM = meropenem; TN = tobramycin; ATM = aztreonam; NA = nalidixic acid CPM = cefepime; E = erythromycin; VA = vancomycin; C = chloramphenicol; RF = rifampin MDR multidrug resistant: non-susceptible to at least one agent in three antimicrobial categories, S = sensitive; I = intermediate; R = resistant.

#### Incidence of MDR, XDR, and PDR strains

The antimicrobial susceptibility profiles of 86 bacterial isolates were identified. From the detected bacterial strains, 53(61.6%) bacterial strains were MDR, 10 (11.6%) were XDR, and 2 (2.3%) were PDR. Among the tested gram-negative isolates, 10/68(14.71%) were XDR, and 2/68(2.94%) were PDR. Almost all of the XDR was attributed to *E*. *coli* (8). However, the incidence of XDR and PDR strains was not observed in all identified gram-positive bacteria (Table 7).

**Table 7:** Phenotypic resistance profile of bacteria isolated from the urine of under-five children at HFSUH, eastern Ethiopia, 2021.

*Bacterial isolates*	*level of resistance*, *n (%)*											
	R0	R1	R2	R3	R4	R5	R6	R7	R≥8	MDR	XDR	PDR
***E*. *coli* (28*)***	4	1	3	1	2	4	2	3	8	20(71.43)	8(28.57)	1(3.57)
***K*. *pneumoniae* (13)**	1	1	2	1	2	0	2	2	2	9(69.23)	1(7.69)	1(7.69)
***Citrobacter* spp. (8)**	0	0	0	0	1	1	2	3		8(100)	1(12.5)	0
***P*. *aeruginosa* (5)**	1	1	2	1	0	0	0	0	0	1(20)	0	0
***E*. *aerogenes* (3)**	0	0	1	0	0	1	1	0	0	2(66.67)	0	0
**S. Paratypi A (3)**	0	0	0	0	0	1	0	1	1	3(100)	0	0
***K*. *oxytoca* (2)**	0	0	1	0	0	0	1	0	0	1(50)	0	0
***M*.*morganii* (2)**	0	1	0	0	0	1	0	0	0	1(50)	0	0
***K*. *ozaenae /K*. *rhinoscleromatis (*1)**	0	0	0	0	0	0	0	1	0	1	0	0
***T*. *ptyseos (*1)**	0	0	1	0	0	0	0	0	0	0	0	0
***Providencia* spp. (2)**	0	0	1	0	0	1	0	0	0	1(50)	0	0
**Total (68)**	6	4	11	3	5	9	8	10	12	47(69.12)	10(14.71)	2(2.94)
***S*. *aureus* (6)**	1	1	1	0	0	0	1	2	0	3(50)	0	0
***S*. *epidermidis* (3)**	0	1	1	0	0	0	1	0	0	1(33.33)	0	0
***S*. *saprophyticus* (2)**	0		1	0	0	0	1	0	0	1(50)	0	0
***Enterococcus* spp. (5)**	0	3	1	0	0	1	0	0	0	1(20)	0	0
***S*. *pyogenes* (2)**	0	2		0	0	0	0	0	0	0	0	0
**Total (18)**	1	7	4	0	0	1	3	2	0	6(33.33)	0	0

Keys: R0: sensitive for all classes of antibiotics, R1: resistant for one class of antibiotics, R2: resistant for two classes of antibiotics, R3: resistant for three classes of antibiotics, etc., MDR-multidrug resistant, XDR-extreme drug-resistant, PDR-pan drug-resistant [26]

## Discussion

Although UTIs can cause serious illness in children, the definitive diagnosis of UTIs is often overlooked in most developing countries due to a lack of facilities, high laboratory costs, and the challenges of obtaining urine from children, especially those who would not void voluntarily [14]. Presently, the effectiveness of commonly prescribed drugs is decreasing globally due to increasing resistant strains. As a result, an infection caused by those strains is challenging; it becomes difficult to treat [5, 36]. Colony counts below 10^5^ CFU/mL in random, clean urine samples for toilet trained children and below 5 x 10^4^ CFU/mL for children under 2 years old and catheterized or SPA urine are not considered significant for defining a UTI [37]; however, this cannot be ignored in patients with immunosuppressed.

In this study, the overall prevalence of bacterial UTIs in children was 24.1%, which is in agreement with other studies in Hawassa, Ethiopia (27.5%) [14], Uganda (26.8%) [27], and Tanzania (20.65%) [38]. However, this finding was higher than studies conducted in Bahirdar, Ethiopia (16.7%) [15], Abakaliki, Nigeria (3%) [39], Enugu, Nigeria (11%) [13], and Dar es Salaam, Tanzania (16.7%) [40]. The disparity between studies could result from the difference in nutritional status, immunity of the population, and method of lab diagnosis [41]. Other explanations could be variations in sample size, the definition of bacteriuria, and the duration of the study.

This study’s most common isolate was *Escherichia coli* (28.75%), which is comparable to some studies of Ethiopia [14, 15]. *E*. *coli* is the most common organism identified in nearly all UTI studies worldwide: 71.7% [13], 31.8% [38], 35.7% [42], and 41.2% [43]. The high rate of *E*. *coli* might be due to the high abundance of *E*. *coli* in the fecal flora, which ascends through the genitalia to cause UTI, in addition to having a unique structure such as P-fimbriae or pili adherence factors, which promote *E*. *coli* attachment to the uroepithelial cells, allowing for multiplication and tissue invasion [44]. But, this finding contradicts a study done in Abakaliki, Nigeria in which *Klebsiella* spp. (24.5%) were most commonly isolated [39], and in Uganda, *Proteus* spp. (39.5%) were most frequently isolated [27]. This could be due to variations in specimen collection techniques and the existence of many virulence factors [44–46].

On the analysis, being a rural resident was significantly associated with bacterial UTIs (p = 0.008) because the majority of study participants were from rural settings and host fecal flora might be the source of the infections [47]. Boys who were not circumcised had a nearly four-fold higher risk of UTIs. This could be because men with uncircumcision and obstructive uropathies have a higher risk of UTIs. As a result, a tight foreskin may interfere with the normal passage of urine and hinder the full emptying of the bladder [48]. patients who self-medicated without prescription were 7 times more likely to acquire UTIs; this finding contrast with another study conducted in Hawassa, Ethiopia [14]. These variations could be attributed to the low rate of self-medication in Hawassa (5.9%) when compared to this study’s (12%).

The history of previous UTIs shows six times more susceptibility to culture-positive results than the correlative one, similar to earlier studies in Bahirdar, Ethiopia [15]. It may be caused by the persistence of resistant strains from previous uropathogenic infections or by the recurrence of those illnesses [49]. Patients with indwelling catheterization have an eleven times higher risk of developing catheter associated UTIs than the non-indwelling catheterized group, which is contrary to the study in Gondar, Ethiopia [47]. Due to the presence of an indwelling catheter device and potentially pathogenic multidrug-resistant organisms in the hospital environment, catheter insertion predisposes to the development of catheter-associated UTIs by creating a new entry point for bacterial invasion and pushing bacteria into the bladder [50]. On the analysis, diabetic patients were an independent risk factor for the acquisition of bacterial UTIs when compared with non-diabetic patients (p = 0.014). This might be due to frequent urination and a high blood sugar level because the high sugar level gives a favorable growth environment to the pathogens [51]. Study participants with a body temperature of ≥ 38.5°C had 4 times more risk for bacterial UTI compared to their counterparts; this result is consistent with the study done in Tanzania [12]. Though it was contradicted by the study in Nigeria [52]. It could be due to geographical variation, climatic change, and differences in the number of study participants. Under-five children with symptoms of urinary frequency were six times more likely to have a culture-positive result. This finding was a disparity from the study in Nigeria [13]. Frequently urinating, infections, injuries, or irritation of the bladder, as well as changes in the muscles and nerves that regulate bladder function, could all be to blame for these variances [53].

Healthcare personnel’s ability to choose proper treatment for UTI has been affected by growing antibiotic resistance [54]. In the present study, meropenem (92.1%), ciprofloxacin (83.3%), and amoxicillin-clavulanic acid (75%) in the carbapenem, fluoroquinolone, and aminoglycoside classes of antibiotics, respectively, were more effective for gram-negative isolates. However, β-lactam antibiotics (ampicillin (10%), cefazolin (27%), and ceftazidime (32%)), tetracycline (22%), and trimethoprim-sulfamethoxazole (28%) were least effective for gram-negative pathogens. The increased rate of beta-lactam and other antibiotic resistance was probably associated with the continuous use of these drugs without prescription or susceptibility data, easy availability, limited diagnostic facilities, the tendency of a patient to use relatively low-cost drugs for all infections, and misuse of antibiotics [15, 55]. Contradictory results from a study in Gusau, Nigeria, showed susceptibility to Gentamycin (63.8%) and Nitrofurantoin (59.6%) [1]. It might be due to the gradual increase in drug resistance and poor adherence [29].

In this study, meropenem (92.8%), amoxicillin-clavulanic acid (75%), and ciprofloxacin (71.4%) were more effective for *E*. *coli*, as with some studies in Bahirdar, Ethiopia [15], Nigeria [1], and Tanzania [38], but it was resistant to the commonly prescribed penicillin drugs (ampicillin (82%)), cephalosporin antibiotics (cefazolin (75%), and ceftazidime (67.8%)), tetracycline (82.1%), and trimethoprim-sulfamethoxazole (75%). A similar finding has been reported from Bahirdar, Ethiopia [15], Hawassa, Ethiopia [14], Tanzania [40], and Kenya [29]. Some studies have found that trimethoprim-sulphamethoxazole, ceftazidime, and augmentin are more effective against *E*. *coli* [1, 24, 38, 47]. It is probably due to multiple factors, including recurrent UTIs, inappropriate prescribing, poor hygiene or fecal colonization, and the presence of extended-spectrum beta-lactamase (ESBL) producing *E*. *coli* [55–57]. In addition, drug resistance can exist naturally, such as through efflux pumps, alteration of the drug-binding site, membrane permeability, or degradation enzymes [36].

*S*. *aureus* was more resistant to the commonly prescribed beta-lactam antibiotics like ampicillin (83.3%) and tetracycline (83.3%), a similar finding was reported from other studies [14, 29], whereas tetracycline and chloramphenicol showed low resistance for *S*. *aureus* in the study from Gondar, Ethiopia [47]. This discordance could be due to *S*. *aureus* producing excessive β-lactamase, and acquiring resistance through plasmid-mediated transduction, transformation, and the insertion of drug-resistant genes of *S*.*aureus* [58].

Furthermore, the overall MDR rate of isolates in our study was 61.6% (95% CI: 51–73, N = 53). Specifically, gram-negative bacterial isolates showed a significant level of MDR 47/68 (69.1%) compared to gram-positive bacteria 6/18 (33.3%), in conjunction with others from Ethiopia [15, 24, 47]. the present study, 11.6% and 2.3% of the gram-negative species were XDR and PDR, respectively. Contradictory results from a study in Ethiopia for 22% XDR and 4% PDR [5] were observed. The use of broad-spectrum agents, increasing irrational antibiotics use, the easy availability of antimicrobials in non-controlled pharmacies, the transmission of resistance genes between people or between people and animals, and over-prescription are all factors that enhance the growth of resistance [5, 15]. And extremely high bacterial resistance in the general population, resulting in overuse of high-potency antibiotics for UTIs, thus increasing bacterial resistance. Antimicrobial resistance among uropathogens is alarmingly increasing with different resistance mechanisms [59, 60]. The commonest acquired antibiotic resistance mechanisms are: preventing the uptake of antibiotic agents (turning off the production of porin channel proteins), modifying a drug target, inactivating a drug through the production of enzymes, and enhancing drug efflux pumps [61]. *E*. *coli* isolate was the most common antibiotic-resistant pathogen in this study; it may produce a wide variety of β-lactamases, including ESBLs, penicillinase and carbapenemases, modification of lipopolysaccharides (LPS), and efflux pumps [62–64]. *K*. *pneumoniae* becomes resistant to commonly available drugs through the development of various first-generation β-lactamases (which are naturally resistant to penicillin); through the making of broad spectrum β-lactamases; through the yielding of ESBLs or penicillinases; and through non-carbapenemase mechanisms of resistance such as the turn of outer membrane proteins and the occurrence of efflux pumps, which can also act harmoniously with the overexpression of β-lactamases [63]. *S*. *aureus* has been able to develop resistance mechanisms to most antibiotics used against it through modification of penicillin-binding protein-2a (PBP2a), penicillinase, ribosomal methylation of binding sites, and efflux pumps. Similar to all β-lactam medications, *S*. *aureus* gained methicillin resistance through the development of PBP2a, which is resistant to all β-lactam antibiotics [65, 66].

### Limitations of the study

One major limitation is the inability to identify some bacterial isolates into species and serotypes. In additions, that the possibility of contamination (especially of gram-negative organisms) exists in the bag or open diaper collection methods. Furthermore, the study could not indicate the molecular characteristics, ESBL and CRE of isolates due to a lack of resources.

## Conclusion and recommendation

In comparison to the majority of other investigations conducted in Africa, more than one-fourth of the children had cultures that were positive for various bacterial uropathogens. UTIs were made more common by indwelling catheterization, a prior history of the condition, males who had not undergone circumcision, a history of using antibiotics without a prescription, rural dwellers, diabetes mellitus, children’s body temperatures, and urine frequency. Both gram-positive species and a variety of gram-negative species were documented, which yielded 24%. The most common isolates that cause UTIs are *E*. *coli*, *K*. *pnemoniae*, and *S*. *aureus*. These strains showed different percentages of susceptibility to the tested antibiotics. The current study points out that meropenem, ciprofloxacin, and amoxicillin-clavulanic acid are choices of drugs for UTI in the study area. Significantly high proportions of MDR strains were observed, especially against commonly used drugs. As patients are diagnosed and treated empirically in the study setting, hospitals should establish antimicrobial stewardship programs. Patients should be managed with microbiological evidence to avoid antimicrobial prescriptions for patients without bacterial infection and better control the spread of drug resistance. The careful prescribing of antibiotics is needed to halt the progress of drug resistance. Hence Working on the identified associated factors is vital to minimize the observed high prevalence of UTIs.

## Supporting information

S1 Data set Dataset used for the analysis of antimicrobial susceptibility result (SPSS). (SAV)

S2 Data set Supplementary material. (SAV)

S3 Data set Supplemental material for Bacterial profile, their antibiotic susceptibility pattern, and associated factors of urinary tract infections in children at Hiwot Fana specialized university hospital, Eastern Ethiopia. (XLSX)

## Abbreviations and acronyms

AST: Antimicrobial Susceptibility Test
ATCC: American Type Culture Collection
API: Analytical Profile Index
CFU: Colony Forming Units
CLED: Cysteine Lactose Electrolyte Deficient
CLSI: Clinical Laboratory Standards Institute
CoNS: Coagulase Negative *Staphylococci*
ECDC: European Centre for Disease Prevention and Control
ESBLs: Extended-spectrum Beta-Lactamases
HFSUH: Hiwot Fana Specialized University Hospital
KIA: Kligler Iron Agar
MDR: Multi-Drug Resistance
PBP2a: Penicillin-Binding Proteins 2a
PDR: Pan Drug Resistance
SOP: Standard Operating Procedures
SPSS: Statistical Package for the Social Sciences
UTI: Urinary Tract Infections
WHO: World Health Organization
XDR: Extremely Drug Resistance

## References

[1] Garba GIBI, Aminu MS, Onazi SO, Yusuf I, Adelakun MB, Kolawole T. Childhood urinary tract pathogens and antibiotic susceptibility seen at Gusau, Nigeria. Tropical Journal of Nephrology. 2015;10(1):7–12.

[2] Flores-Mireles AL, Walker JN, Caparon M, Hultgren SJ. Urinary tract infections: epidemiology, mechanisms of infection and treatment options. Nature reviews microbiology. 2015;13(5):269–84. doi: 10.1038/nrmicro3432 25853778 PMC4457377

[3] Faidah HS, Ashshi AM, Abou El-Ella GA, Al-Ghamdi AK, Mohamed AM. Urinary tract infections among pregnant women in Makkah, Saudi Arabia. Biomedical And Pharmacology Journal. 2015;6(1):01–7.

[4] Sibi G, Devi AP, Fouzia K, Patil BR. Prevalence, microbiologic profile of urinary tract infection and its treatment with trimethoprim in diabetic patients. Research journal of microbiology. 2011;6(6):543.

[5] Addis T, Mekonnen Y, Ayenew Z, Fentaw S, Biazin H. Bacterial uropathogens and burden of antimicrobial resistance pattern in urine specimens referred to Ethiopian Public Health Institute. PloS one. 2021;16(11):e0259602. doi: 10.1371/journal.pone.0259602 34767605 PMC8589166

[6] Ranganathan V. Urinary tract infection: an overview of the infection and the associated risk factors. Journal of Microbiology & Experimentation. 2014;1(2):3–5.

[7] Sewify M, Nair S, Warsame S, Murad M, Alhubail A, Behbehani K, et al. Prevalence of urinary tract infection and antimicrobial susceptibility among diabetic patients with controlled and uncontrolled glycemia in Kuwait. Journal of diabetes research. 2016;2016. doi: 10.1155/2016/6573215 26844231 PMC4710901

[8] Hailay A, Zereabruk K, Mebrahtom G, Aberhe W, Bahrey D. Magnitude and Its Associated Factors of Urinary Tract Infection among Adult Patients Attending Tigray Region Hospitals, Northern Ethiopia, 2019. International Journal of Microbiology. 2020;2020. doi: 10.1155/2020/8896990 32774382 PMC7407062

[9] Mashouf RY, Babalhavaeji H, Yousef J. Urinary tract infections: bacteriology and antibiotic resistance patterns. Indian pediatrics. 2009;46(7). 19430071

[10] Edlin RS, Shapiro DJ, Hersh AL, Copp HL. Antibiotic resistance patterns of outpatient pediatric urinary tract infections. The Journal of urology. 2013;190(1):222–7. doi: 10.1016/j.juro.2013.01.069 23369720 PMC4165642

[11] Sargiary P, Baro L, Choudhry G, Saikia L. Bacteriological profile and antimicrobial susceptibility pattern of community acquired urinary tract infection in children: a tertiary care experience. J Dental Med Sci. 2016;15(6):61–5.

[12] Msaki BP, Mshana SE, Hokororo A, Mazigo HD, Morona D. Prevalence and predictors of urinary tract infection and severe malaria among febrile children attending Makongoro health centre in Mwanza city, North-Western Tanzania. Archives of Public Health. 2012;70(1):4. doi: 10.1186/0778-7367-70-4 22958592 PMC3415110

[13] Ibeneme C, Oguonu T, Okafor H, Ikefuna A, Ozumba U. Urinary tract infection in febrile under five children in Enugu, South Eastern Nigeria. Nigerian journal of clinical practice. 2014;17(5):624–8. doi: 10.4103/1119-3077.141430 25244275

[14] Mitiku E, Amsalu A, Tadesse BT. Pediatric urinary tract infection as a cause of outpatient clinic visits in southern Ethiopia: a cross sectional study. Ethiopian journal of health sciences. 2018;28(2):187–96. doi: 10.4314/ejhs.v28i2.10 29983516 PMC6016340

[15] Fenta A, Dagnew M, Eshetie S, Belachew T. Bacterial profile, antibiotic susceptibility pattern and associated risk factors of urinary tract infection among clinically suspected children attending at Felege-Hiwot comprehensive and specialized hospital, Northwest Ethiopia. A prospective study. BMC Infectious Diseases. 2020;20(1):1–10. 10.1186/s12879-020-05402-y PMC7493977 32938424

[16] WHO. Anti microbial risistance 2020.

[17] McLellan LK, Hunstad DA. Urinary tract infection: pathogenesis and outlook. Trends in molecular medicine. 2016;22(11):946–57. doi: 10.1016/j.molmed.2016.09.003 27692880 PMC5159206

[18] Basak S, Singh P, Rajurkar M. Multidrug resistant and extensively drug resistant bacteria: a study. Journal of pathogens. 2016;2016. doi: 10.1155/2016/4065603 26942013 PMC4749793

[19] Rosenberger LH, Hranjec T, Politano AD, Swenson BR, Metzger R, Bonatti H, et al. Effective cohorting and “superisolation” in a single intensive care unit in response to an outbreak of diverse multi-drug-resistant organisms. Surgical infections. 2011;12(5):345–50. doi: 10.1089/sur.2010.076 21936667 PMC4845630

[20] Shume T, Tesfa T, Mekonnen S, Asmerom H, Tebeje F, Weldegebreal F. Aerobic Bacterial Profile and Their Antibiotic Susceptibility Patterns of Sterile Body Fluids Among Patients at Hiwot Fana Specialized University Hospital, Harar, Eastern Ethiopia. Infection and Drug Resistance. 2022;15:581. doi: 10.2147/IDR.S351961 35228808 PMC8882023

[21] Marami D, Balakrishnan S, Seyoum B. Prevalence, Antimicrobial Susceptibility Pattern of Bacterial Isolates, and Associated Factors of Urinary Tract Infections among HIV‐Positive Patients at Hiwot Fana Specialized University Hospital, Eastern Ethiopia. Canadian Journal of Infectious Diseases and Medical Microbiology. 2019;2019. doi: 10.1155/2019/6780354 30881531 PMC6381576

[22] Tesfa T, Baye Y, Sisay M, Amare F, Gashaw T. Bacterial uropathogens and susceptibility testing among patients diagnosed with urinary tract infections at Hiwot Fana Specialized University Hospital, Eastern Ethiopia. SAGE open medicine. 2021;9:20503121211001162. doi: 10.1177/20503121211001162 33796299 PMC7970184

[23] Belete Y, Asrat D, Woldeamanuel Y, Yihenew G, Gize A. Bacterial profile and antibiotic susceptibility pattern of urinary tract infection among children attending Felege Hiwot Referral Hospital, Bahir Dar, Northwest Ethiopia. Infection and drug resistance. 2019:3575–83. doi: 10.2147/IDR.S217574 31819542 PMC6874112

[24] Merga Duffa Y, Terfa Kitila K, Mamuye Gebretsadik D, Bitew A. Prevalence and Antimicrobial Susceptibility of Bacterial Uropathogens Isolated from Pediatric Patients at Yekatit 12 Hospital Medical College, Addis Ababa, Ethiopia. International journal of microbiology. 2018. doi: 10.1155/2018/8492309 30386381 PMC6189692

[25] Mishra MP, Sarangi R, Padhy RN. Prevalence of multidrug resistant uropathogenic bacteria in pediatric patients of a tertiary care hospital in eastern India. Journal of infection and public health. 2016;9(3):308–14. doi: 10.1016/j.jiph.2015.10.002 26617250

[26] Magiorakos A-P, Srinivasan A, Carey RB, Carmeli Y, Falagas M, Giske C, et al. Multidrug-resistant, extensively drug-resistant and pandrug-resistant bacteria: an international expert proposal for interim standard definitions for acquired resistance. Clinical microbiology and infection. 2012;18(3):268–81. doi: 10.1111/j.1469-0691.2011.03570.x 21793988

[27] Onzima RAD, Ocokoru C, Govule P. Predictive validity and reliability of dipstick and microscopy in diagnosis of urinary tract infections among febrile under-fives in Nsambya hospital, Uganda. Open Science Journal of Clinical Medicine. 2015;3(3):107.

[28] Kadigi D, Mosha F, Moyo S, Matee M. Etiology and Antimicrobial Susceptibility Patterns of Bacterial Agents Causing Urinary Tract Infection in Children under Five years, dar es Salaam. Journal of Biotechnology and Immunology. 2020;2(1):2.

[29] Masika WG, O’Meara WP, Holland TL, Armstrong J. Contribution of urinary tract infection to the burden of febrile illnesses in young children in rural Kenya. PLoS One. 2017;12(3):e0174199. doi: 10.1371/journal.pone.0174199 28323886 PMC5360311

[30] Strasinger SK, Di Lorenzo MS. Urinalysis and body fluids: FA Davis; 2014.

[31] Cheesbrough M. District laboratory practice in tropical countries, part 2: Cambridge university press; 2005.

[32] Mehboob M, Hakim M, Ullah O, Lodhi SS, Anees M, Khalil I, et al. Identification and Characterization of Urinary Tract Infectious Bacteria and its Antibiotic Sensitivity. BioScientific Review. 2021;3(3):43–62.

[33] AL-Joda BMS JA. Biochemical testing revision for identification several kinds of bacteria. JUBPAS. 2021;29(2):168–76.

[34] CLSI. Performance Standards for Antimicrobial Susceptibility Testing. M100, 30th ed. 2020; 40(1).

[35] Zhang Z. Model building strategy for logistic regression: purposeful selection. Annals of translational medicine. 2016;4(6). doi: 10.21037/atm.2016.02.15 27127764 PMC4828741

[36] Chokshi A, Sifri Z, Cennimo D, Horng H. Global contributors to antibiotic resistance. Journal of global infectious diseases. 2019;11(1):36. doi: 10.4103/jgid.jgid_110_18 30814834 PMC6380099

[37] Primack W, Bukowski T, Sutherland R, Gravens-Mueller L, Carpenter M. What urinary colony count indicates a urinary tract infection in children? The Journal of pediatrics. 2017;191:259–61. e1. doi: 10.1016/j.jpeds.2017.08.012 28967387 PMC5705283

[38] Ahmed M, Moremi N, Mirambo MM, Hokororo A, Mushi MF, Seni J, et al. Multi-resistant gram negative enteric bacteria causing urinary tract infection among malnourished underfives admitted at a tertiary hospital, northwestern, Tanzania. Italian Journal of Pediatrics. 2015;41(1):1–5. 26084628 10.1186/s13052-015-0151-5 PMC4472394

[39] Muoneke V, Ibekwe M, Ibekwe R. Childhood urinary tract infection in abakaliki: etiological organisms and antibiotic sensitivity pattern. Annals of medical and health sciences research. 2012;2(1):29–32. doi: 10.4103/2141-9248.96933 23209987 PMC3507128

[40] Fredrick F, Francis JM, Fataki M, Maselle SY. Aetiology, antimicrobial susceptibility and predictors of urinary tract infection among febrile under-fives at Muhimbili National Hospital, Dar es Salaam-Tanzania. African journal of microbiology research. 2013;7(12):1029–34.

[41] Okwara F, Obimbo E, Wafula E, Murila F. Bacteraemia, urinary tract infection and malaria in hospitalised febrile children in Nairobi: is there an association? East African medical journal. 2004;81(1):47–51. doi: 10.4314/eamj.v81i1.8795 15080516

[42] Afsharpaiman S, Bairaghdar F, Torkaman M, Kavehmanesh Z, Amirsalari S, Moradi M, et al. Bacterial pathogens and resistance patterns in children with community-acquired urinary tract infection: a cross sectional study. Journal of Comprehensive Pediatrics. 2012;3(1):16–20.

[43] Francis F, Joel MF, Maulidi F, Samuel YM. Aetiology, antimicrobial susceptibility and predictors of urinary tract infection among febrile under-fives at Muhimbili National Hospital, Dar es Salaam-Tanzania. African journal of microbiology research. 2013;7(12):1029–34.

[44] Schilling JD, Mulvey MA, Hultgren SJ. Structure and function of Escherichia coli type 1 pili: new insight into the pathogenesis of urinary tract infections. The Journal of infectious diseases. 2001;183(Supplement_1):S36–S40. doi: 10.1086/318855 11171011

[45] Adekunle AA, Adetokunbo AS. Prevalence and predictors of asymptomatic bacteriuria in HIV positive pregnant women. Online J Med Med Sci Res. 2014;3(5):48–54.

[46] Butel M-J, Waligora-Dupriet A-J, Wydau-Dematteis S. The developing gut microbiota and its consequences for health. Journal of Developmental Origins of Health and Disease. 2018;9(6):590–7. doi: 10.1017/S2040174418000119 29562949

[47] Ayelign B, Abebe B, Shibeshi A, Meshesha S, Shibabaw T, Addis Z, et al. Bacterial isolates and their antimicrobial susceptibility patterns among pediatric patients with urinary tract infections. Turkish journal of urology. 2018;44(1):62. doi: 10.5152/tud.2017.33678 29484230 PMC5821286

[48] Irkilata L, Aydin HR, Aydin M, Gorgun S, Demirel HC, Adanur S, et al. Preputial bacterial colonisation in uncircumcised male children: Is it related to phimosis. J Pak Med Assoc. 2016;66(3):312–5. 26968283

[49] Hanna-Wakim RH, Ghanem ST, El Helou MW, Khafaja SA, Shaker RA, Hassan SA, et al. Epidemiology and characteristics of urinary tract infections in children and adolescents. Frontiers in cellular and infection microbiology. 2015;5:45. doi: 10.3389/fcimb.2015.00045 26075187 PMC4443253

[50] Gebremariam G, Legese H, Woldu Y, Araya T, Hagos K, GebreyesusWasihun A. Bacteriological profile, risk factors and antimicrobial susceptibility patterns of symptomatic urinary tract infection among students of Mekelle University, northern Ethiopia. BMC infectious diseases. 2019;19(1):1–11. 31703645 10.1186/s12879-019-4610-2 PMC6842233

[51] Wang M-C, Tseng C-C, Wu A-B, Lin W-H, Teng C-H, Yan J-J, et al. Bacterial characteristics and glycemic control in diabetic patients with Escherichia coli urinary tract infection. Journal of Microbiology, Immunology and Infection. 2013;46(1):24–9. doi: 10.1016/j.jmii.2011.12.024 22572000

[52] Rabasa A, Gofama M. Urinary tract infection in febrile children in Maiduguri North Eastern Nigeria. Nigerian journal of clinical practice. 2009;12(2). 19764657

[53] Parnell BA. Overactive bladder: an urgent problem. Southern medical journal. 2011;104(1):7–8. doi: 10.1097/SMJ.0b013e3181fd2aef 21079531

[54] White B. Diagnosis and treatment of urinary tract infections in children. American family physician. 2011;83(4):409–15. 21322515

[55] Ikeda Y, Mamiya T, Nishiyama H, Koseki T, Mouri A, Nabeshima T. Risk factors for extended-spectrum β-lactamase-producing Escherichia coli infection in hospitalized patients. Nagoya journal of medical science. 2012;74(1–2):105. 22515116 PMC4831255

[56] Aljanaby AAJ, Aljanaby IAJ. Antimicrobial sensitivity pattern of pathogenic bacteria isolated from older women with asymptomatic bacteriuria. Biomedical Research. 2018;29(12):2597–601.

[57] Galindo-Méndez M. Antimicrobial resistance in Escherichia coli. E Coli Infections-Importance of Early Diagnosis and Efficient Treatment. 2020:1–20.

[58] Guo Y, Song G, Sun M, Wang J, Wang Y. Prevalence and therapies of antibiotic-resistance in Staphylococcus aureus. Frontiers in cellular and infection microbiology. 2020;10:107. doi: 10.3389/fcimb.2020.00107 32257966 PMC7089872

[59] Devrim F, Serdaroğlu E, Çağlar İ, Oruç Y, Demiray N, Bayram N, et al. The emerging resistance in nosocomial urinary tract infections: from the pediatrics perspective. Mediterranean journal of hematology and infectious diseases. 2018;10(1). doi: 10.4084/MJHID.2018.055 30210748 PMC6131100

[60] Gniadkowski M. Evolution and epidemiology of extended-spectrum β-lactamases (ESBLs) and ESBL-producing microorganisms. Clinical Microbiology and Infection. 2001;7(11):597–608. 11737084 10.1046/j.1198-743x.2001.00330.x

[61] Reygaert WC. An overview of the antimicrobial resistance mechanisms of bacteria. AIMS microbiology. 2018;4(3):482. doi: 10.3934/microbiol.2018.3.482 31294229 PMC6604941

[62] Al-Agamy MH, Aljallal A, Radwan HH, Shibl AM. Characterization of carbapenemases, ESBLs, and plasmid-mediated quinolone determinants in carbapenem-insensitive Escherichia coli and Klebsiella pneumoniae in Riyadh hospitals. Journal of infection and public health. 2018;11(1):64–8. doi: 10.1016/j.jiph.2017.03.010 28462854

[63] Kakoullis L, Papachristodoulou E, Chra P, Panos G. Mechanisms of antibiotic resistance in important gram-positive and gram-negative pathogens and novel antibiotic solutions. Antibiotics. 2021;10(4):415. doi: 10.3390/antibiotics10040415 33920199 PMC8069106

[64] Poirel L, Madec J-Y, Lupo A, Schink A-K, Kieffer N, Nordmann P, et al. Antimicrobial resistance in Escherichia coli. Microbiology Spectrum. 2018;6(4):6.4. 14. 10.1128/microbiolspec.arba-0026-2017 PMC11633601 30003866

[65] Foster TJ. Antibiotic resistance in Staphylococcus aureus. Current status and future prospects. FEMS microbiology reviews. 2017;41(3):430–49. doi: 10.1093/femsre/fux007 28419231

[66] Moisan H, Pruneau M, Malouin F. Binding of ceftaroline to penicillin-binding proteins of Staphylococcus aureus and Streptococcus pneumoniae. Journal of antimicrobial chemotherapy. 2010;65(4):713–6. doi: 10.1093/jac/dkp503 20097788

